# Expanding the Concept of G Protein-Coupled Receptor (GPCR) Dimer Asymmetry towards GPCR-Interacting Proteins

**DOI:** 10.3390/ph4020273

**Published:** 2011-01-25

**Authors:** Maud Kamal, Pascal Maurice, Ralf Jockers

**Affiliations:** 1 Institut Cochin, Universite Paris Descartes, CNRS, Paris, France; 2 Inserm, U1016, Paris, France; E-Mails: maud.kamal@inserm.fr (M.K.); pascal.maurice@inserm.fr (P.M.)

**Keywords:** GPCR, dimerization, GIP, allosterism

## Abstract

G protein-coupled receptors (GPCRs), major targets of drug discovery, are organized in dimeric and/or oligomeric clusters. The minimal oligomeric unit, the dimer, is composed of two protomers, which can behave differently within the dimer. Several examples of GPCR asymmetry within dimers at the level of ligand binding, ligand-promoted conformational changes, conformational changes within transmembrane domains, G protein coupling, and most recently GPCR-interacting proteins (GIPs), have been reported in the literature. Asymmetric organization of GPCR dimers has important implications on GPCR function and drug design. Indeed, the extension of the “asymmetry concept” to GIPs adds a new level of specific therapeutic intervention.

## 1. Introduction

A growing body of pharmacological, biochemical and biophysical data indicate that GPCRs form functional homo- and hetero-dimers and most likely higher-order oligomers [1,2]. Formation of such oligomeric structures has been shown to provide shielding from the quality-control checkpoints during the biosynthetic pathway [3] and to have an important role in modulating GPCR function and signaling [4]. In addition, this oligomeric organization raises a fundamental question: does each protomer within a GPCR oligomer operate as an independent functional unit or do the different protomers intercommunicate to insure the signaling response of the cell? Communication within dimers has indeed been observed for several GPCRs at different levels. Ligand binding to one protomer has been shown to modify the properties of the ligand binding pocket of the second protomer. The latter observation most likely involves ligand-induced conformational changes that are transmitted from one protomer to the other. Intercommunication among the different protomers results in dimer asymmetry and asymmetric recruitment of GPCR-interacting proteins (GIPs) including heterotrimeric G proteins. This mini-review summarizes the evidence supporting the emerging concept of asymmetric behavior within a GPCR complex. We will briefly discuss asymmetry in G protein coupling, ligand binding, conformational changes within transmembrane domains, and binding of GIPs.

## 2. Asymmetry at the Level of G Protein Binding

**Figure 1:**
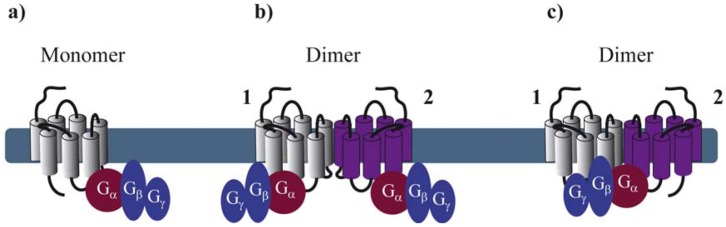
Stoichiometry of G protein coupling to GPCR dimers. **(a)** The minimal functional unit is constituted of one monomeric receptor and one heterotrimeric G protein; **(b)** Two G proteins binding, each, one protomer within a dimer; **(c)** Asymmetric G protein coupling to a receptor dimer: only one G protein binds one protomer of the dimer.

Reconstitution of purified monomeric GPCRs, in high-density lipoprotein phospholipid bilayer particles, showed that monomeric GPCRs are able to couple to G proteins. These and similar results clearly demonstrate that the minimal functional unit consists of 1 receptor monomer activating its cognate G protein (Figure 1a) [5-7]. When rod outer segment membranes were solubilized with different detergents to obtain preparations enriched in monomeric, dimeric and oligomeric rhodopsin structures, monomeric rhodopsin was indeed able to activate its G protein, the heterotrimeric transducin (G_t_) confirming studies in reconstituted systems [8]. However, preparations containing mostly dimeric and oligomeric rhodopsin appeared to be more active.

By extrapolating the idea that a GPCR monomer couples to a single G protein, one might anticipate that GPCR dimers, composed of two protomers, bind to two G proteins (Figure 1b). Nevertheless, to our knowledge, there is no clear experimental evidence today confirming such an arrangement, except one study published in 2008 by Parker *et al.* on the neuropeptide Y (NPY) Y2 receptor. Using radiolabeled-ligand binding assays, GTPγS binding and gradient ultracentrifugation experiments, the authors show that Y2 receptors and G proteins could be solubilized in complexes of 2:2, 2:1 and 1:1 stoichiometry, depending on the detergent and the concentration of agonist used [9]. Cumulative evidence from the literature is, however, rather consistent with a 2:1 stoichiometry with two receptor protomers coupling only one heterotrimeric G protein (Figure 1c). Such a stoichiometry implies that GPCR dimers have a radically different mode of function than monomers therefore introducing the concept of asymmetry.

GPCR asymmetry was intensely debated when the first high-resolution structure of rhodopsin, the archetypal class A GPCR, was solved by Palczewski and associates [10]. Computational docking simulations and molecular modeling of rhodopsin and its G_t_ protein illustrated that the receptor-facing surface of G_t_ is too large with respect to the rhodopsin cytoplasmic surface. These results suggest that GPCR dimer formation is a pre-requisite to fully accommodate the binding of the G protein [11,12]. Concomitantly, a 2:1 stoichiometry was proposed for another class A GPCR, the leukotriene B4 receptors BLT_1_ and its cognate G protein, by using purified proteins and chemical cross-linking [13].

These pioneering studies raised the fundamental question of the respective role of each protomer within a dimer in regards to G protein activation. Indeed, there are at least two ways for the G protein to contact the dimer (through protomer 1 or 2). Based on this assumption, asymmetry of the receptor dimer might be dictated by the position of the Gα subunit relative to the agonist-occupied monomer. A possible model would be that activation of one of the protomers favors an oriented interaction of the receptor dimer with the G protein in such a way that the agonist-occupied protomer directly interacts with the G protein and is therefore stabilized in a fully active, high affinity conformation, whereas the other protomer remains in an uncoupled, low affinity conformation [14]. Further important progress on how the receptor dimer interacts with the G protein has been made using BLT_1_ and dopamine D_2_ homodimers as models. By using the intrinsic fluorescence properties of the BLT_1_ receptor to monitor its activation, Damian *et al.* showed that a receptor dimer with only a single agonist-occupied subunit can trigger G protein activation. Interestingly, the two subunits of the dimer in the G protein-coupled state differ in their conformation, even when both protomers are occupied by agonists. In the absence of G protein coupling, no such asymmetric conformational changes were observed [15]. These results suggest that the interaction of the G protein with the receptor dimer would bring specific constraints, which prevent a symmetric functioning of the dimer and that the G protein itself might be partly responsible for the asymmetric functioning in a context where ligand binding *cis*-activates G protein binding to the same protomer [14]. On the other hand, using an original approach based on a functional complementation assay with the D_2_ receptor, allosteric communication between protomers of GPCR dimers was proposed [16]. This study confirmed that agonist binding to a single protomer within the dimer can maximally activate the G protein. Moreover, activation of the second protomer inhibits the functional response whereas inverse agonist binding enhances signaling, definitely proving that the way the two protomers contribute to the activated complex with the G protein is not symmetric, and that activation requires different conformational changes in each protomer [16]. Such asymmetry also applies to class C GPCRs as exemplified by the GABA_B_ receptor, which is an obligatory heteromer composed of GB1 and GB2 subunits, with GB1 binding the ligand and GB2 activating the G protein [17,18].

Collectively, the most convincing examples in the literature favor a 2:1 stoichiometry between the receptor and the G protein. Several studies using purified and reconstituted GPCRs suggest a 1:1 stoichiometry. However, one should be careful when extrapolating these *in vitro* observations into a more physiological context; *i.e.* the plasma membrane of mammalian cells. Asymmetric behavior of GPCR dimers is further supported by observations at the level of ligand binding and ligand-promoted conformational changes within the heptahelical transmembrane domain as discussed in the next paragraphs.

## 3. Asymmetry at the Level of Ligand Binding

The phenomenon of allostery has been observed in many biological processes, and refers to different, albeit related, mechanisms by which protein function can be regulated and fine-tuned in either a positive or negative direction [19]. In pharmacology, allosteric modulation of a receptor results from the binding of allosteric molecules at a different site (allosteric site) from that of the endogenous ligand (orthosteric site). Allosteric modulators normally induce a conformational change in the receptor resulting in a positive or negative effect on binding affinity and efficacy of ligand binding to the orthosteric site. In the context of GPCR oligomerization, each protomer in the complex possesses an orthosteric binding pocket and one can expect that the orthosteric site of one protomer could exert allosteric effects on the orthosteric site of another protomer [20].

Allosteric interactions between ligand binding sites within a dimeric complex result in either positive or negative cooperativity. This fact raises the question of stoichiometry concerning the number of ligand molecules binding a dimer. The majority of the examples in the literature suggest that negative cooperativity is due to a single ligand molecule binding one protomer of the dimer, whereas positive cooperativity results from two ligand molecules binding, each, one protomer in a dimer. However, exceptions from this rule exist as mentioned below.

**Figure 2:**
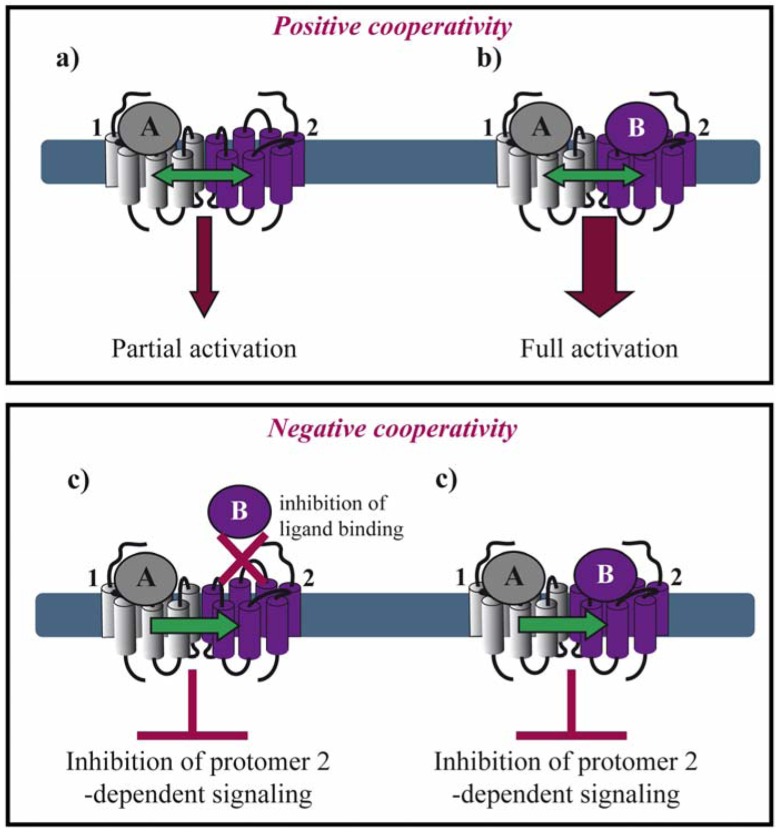
Asymmetry in ligand binding within a GPCR dimer. Positive cooperativity: **(a)** One agonist (A) binding to a dimer results in GPCR partial activation; but **(b**) two agonists (A, B) are required for full activation. Ligand B can be identical to agonist A or a different agonist or an antagonist, which can enhance GPCR activation. Negative cooperativity; **(c)** Agonist A binding to protomer 1 inhibits the binding of ligand B to protomer 2, therefore suppressing protomer 2-dependent signaling; or **(d)** agonist A binding to protomer 1 inhibits ligand B-induced protomer 2 signaling.

Positive cooperativity has early on been reported for several GPCRs. In the case of agonist binding to the μ/δ opioid receptor (OR) heteromer, both agonists are required for efficient MAP kinase activation (Figures 2a,b) [21]. Similarly, activation of both protomers of a muscarinic M3 homomer is required for beta-arrestin recruitment [22]. Positive cooperative binding was also documented for class C GPCRs. Binding of a single agonist is sufficient for the activation of the homomeric metabotropic glutamate receptor (mGluR) 5. However, full activation requires binding of two agonist molecules and the closure of both agonist-binding domains of the homodimer, also called venus fly trap (VFT) [23]. Another type of positive allosterism is seen in the CXCR2/δOR heteromer, where CXCR2 antagonists enhance the function of both peptide and alkaloid-based agonists at the δOR when the two receptors are co-expressed [24].

Negative binding cooperativity of allosteric nature has been observed in other cases such as homo-and heteromers of glycoprotein hormone receptors. Ligand binding to the large extracellular ectodomain triggers activation of constitutive receptor dimers that are mainly stabilized by interactions between the transmembrane domains. Using competition, saturation and dissociation kinetics binding experiments, Urizar *et al.* showed for the first time for class A GPCRs that the two orthosteric sites of each dimer are implicated in negative allosteric interactions with one another (Figure 2c) [25]. Similarly, an extensive pharmacological characterization of the dopamine D_2_ receptor by performing saturation binding assays and dissociation kinetics with three different radioligands showed that some D_2_ receptor antagonists exhibit negative binding cooperativity within a D_2_ homodimer [26]. Furthermore, chemokine receptor heteromers display allosteric modulation in ligand binding and dissociation kinetic experiments strongly suggesting negative cooperativity for several different heteromers. *Trans*-inhibition of ligand binding was observed between CCR5 and CCR2b co-expressed in the same cell line and in cells endogenously expressing both receptors [27,28]. CCR5-specific ligands, unable to compete for the binding of CCR2-specific ligands on cells expressing CCR2 alone, inhibited this binding when both receptors were co-expressed. Similar results were obtained with CCR2-selective ligands.

Moreover, in the context of the μOR/α_2A_-adrenoceptor (AR) heteromer, Vilardaga *et al.* showed that simultaneous binding of the two respective agonists, norepinephrine and morphine, inhibits α_2A_-AR-dependent G_i_ signaling and the downstream MAP kinase cascade [29]. These results interestingly show that negative cooperativity may occur in a dimer where both binding pockets are occupied by their respective agonists (Figure 2d).

Recently, Albizu *et al.* used fluorescent-labeled ligands to study oxytocine receptor dimerization in native tissues. A mixture of two antagonists, either labeled with an energy donor or acceptor, generated a strong FRET signal. However, only marginal FRET signals were obtained when two labeled agonists were used indicating that agonist binding to one protomer suppresses agonist binding to the second protomer whereas no such inhibitory effect is seen for antagonist binding [30]. Both positive and negative cooperativity have also been reported within the same heteromer as illustrated by the dopamine D_2_ receptor and somatostatin sst5 receptor heteromer, for which the positive cooperativity was observed for D_2_ agonists and negative cooperativity for D_2_ antagonists [31].

Collectively, different types of negative and positive allosteric modulations can be observed and may involve binding of one or two agonists or antagonists to the respective GPCR homo- or heteromer. In 2005, Durroux proposed mathematical models attempting to explain the complex allosteric interactions observed between ligand binding sites within dimeric complexes. [32].

## 4. Asymmetry at the Level of Ligand-Induced Conformation Changes

**Figure 3:**
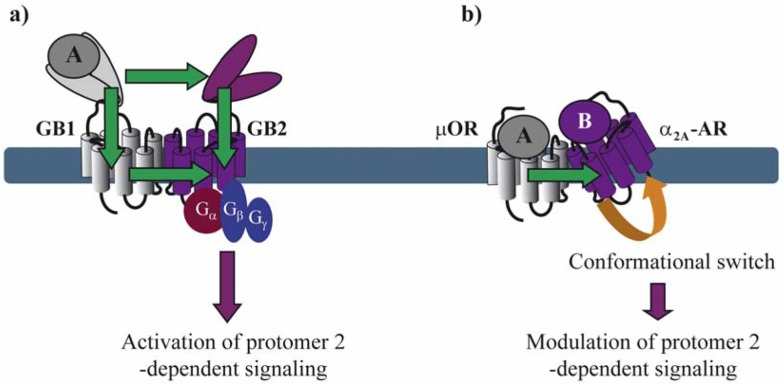
Conformational changes within a GPCR dimer. **(a)** Agonist A binding to the VFT of GB1 induces conformational changes that are transmitted to the VFT and the transmembrane (TM) domain of GB2 and activate its G protein. Binding of agonist A to the VFT of GB1 can also activate the TM domain of GB1 which then *trans*-activates the TM domain of GB2 and its G protein; **(b)** Binding of agonist A to μOR induces a conformational switch in α_2A_-AR resulting in negative modulation of α_2A_-AR-dependent signaling. Panel (a) has been modified from reference 32.

With the exception of neutral antagonists, binding of all other types of ligands ranging from full inverse agonists to full agonists induce conformational changes in GPCRs. Negative and positive cooperativity at the level of ligand binding is further propagated to the subsequent conformational changes within the dimer. Such cross-conformational switches between the two protomers of GPCR dimers have been observed in some cases (Figure 3). In the μOR/α_2A_-AR heteromer, morphine binding to the μOR triggers an inhibitory conformational change with subsecond kinetics in the norepinephrine-occupied α_2A_-AR as monitored directly by FRET in living cells [29].

Asymmetric ligand-induced conformational changes within GPCR dimers have been fully characterized in the GABA_B_ receptor. Within this obligatory heterodimer, GB1 and GB2 subunits accomplish different tasks. The role of the GB2 is to traffic GB1 to the membrane where the VFT of GB1 binds to GABA and *trans*-activates GB2, which in turn couples to G_i_ proteins [33]. By analyzing chimeric and deletion constructs of GB1 and GB2, the authors show that several different conformational changes are necessary for full G protein activation. GABA-induced changes in the relative position of the VFTs of GB1 and GB2 activate the transmembrane (TM) portion of GB2 most likely through two allosteric pathways: (1) one direct from the GB2 VFT to the GB2 TM portion and (2) a second one that interconnects the GB1 VFT to GB1 TM portion, which, in turn, *trans*-activates GB2 TM portion [17,34].

Recently, Damian *et al.* monitored conformational changes within purified BLT_1_ receptor dimers, which were labeled with a single 5-hydroxytryptophan [15]. They showed that a receptor dimer with only a single agonist-occupied subunit can trigger G protein *cis*-activation. Interestingly, asymmetric conformational changes were observed even when both protomers were occupied by the same agonist. This asymmetry was highly dependent on the coupling of the dimer to purified G proteins. Altogether, asymmetric ligand-induced conformational changes have been monitored with several different techniques using different GPCRs either in living cells or as purified proteins.

## 5. Asymmetry at the Level of GPCR-Interacting Proteins (GIPs)

In addition to heterotrimeric G proteins, most GPCRs also bind to other intracellular proteins either directly or indirectly forming large protein complexes as exemplified by the melatonin MT_1_ and MT_2_ receptors [35,36]. Among those proteins that directly interact with GPCRs, GPCR kinases (GRKs) and arrestins are probably the most studied. GPCR signaling is believed to involve the sequential interaction of the activated receptor with the G protein, GRKs and arrestin. In contrast to this current model, recent observations made with energy transfer techniques however suggest that these different events might be interconnected and might overlap in time [37]. Recruitment of GRK2 to the α_2A_-AR, for instance, was shown to occur before dissociation of the G protein, therefore raising the question of the exact molecular organization of this transient protein complex. A first hint of the putative organization of such a complex came from crystallographic data of the GRK2/G_αq_/G_β1γ2_ complex. These data showed that GRK2 can bind G_βγ_ and G_αq_ subunits simultaneously while maintaining a surface for a possible interaction with the receptor [38].

Arrestin is believed to be recruited after the first wave of G protein-dependent signaling events and to compete with G proteins and other GIPs for receptor binding. However, recent data on the parathyroid hormone receptor (PTHR) suggest that at least part of the arrestin binding might be faster than anticipated and occur within a pre-associated complex depending on the GIP Na/H exchange regulatory factor-1 (NHERF1). These results raise once again the question of the composition of GPCR-associated complexes [39].

**Figure 4:**
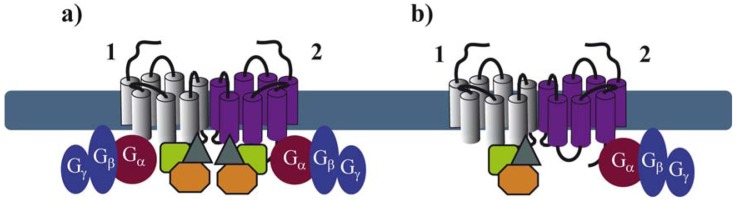
Symmetry **(a)** and asymmetry **(b)** of GIP and G protein coupling within a GPCR dimer.

Recent advances in proteomic approaches for the identification of GPCR-associated protein complexes provided a number of further GIPs that directly or indirectly interact with intracellular domains of GPCRs [40,41]. Among these domains, the C-terminal tail and the third intracellular (i3) loop of the receptor have been shown to be the major interacting domains [42]. Many of these proteins appear to bind constitutively to receptors [43,44]. In order to better understand the exact molecular architecture of these complexes, their precise composition, which might be dynamically regulated in space and time, needs to be determined. It is quite logical to anticipate that GPCRs are likely to bind simultaneously, in a constitutive or a ligand-dependent manner, several GIPs. Binding of these proteins to the extended surface of receptor dimers, offers many possibilities for the spatiotemporal interaction of several GIPs with the same receptor. Some of these interactions may depend on receptor domains that are sufficiently distinct to allow simultaneous binding to the receptor, others are likely to overlap. As illustrated above for G proteins, asymmetric binding of GIPs to GPCR dimers might be a solution to accommodate simultaneous binding of several GIPs (Figure 4). This has been recently illustrated in the ternary complex between the melatonin MT_1_ receptor, G_i_ proteins and the regulator of G protein signaling (RGS) 20 [44]. This study shows that G_i_ and RGS20 are both constitutively binding to overlapping, membrane-proximal domains of MT_1_. These data strongly imply that G_i_ and RGS20 bind to two different protomers within the homodimer.

Similar observations were made for heteromeric complexes of MT_1_ with MT_2_, the second melatonin receptor, suggesting the formation of an asymmetric quaternary complex with MT_1_ binding to RGS20 and MT_2_ binding to G_i_. This example suggests that a receptor, which is not regulated by a specific GIP, could become sensitive to the action of this GIP when heterodimerizing with a GIP-binding receptor. This latter observation thus provides additional options in fine-tuning GPCR function in a context-dependent manner.

## 6. Conclusions and Perspectives

GPCR dimerization is a topic of great interest due to the potential importance of the functional consequences of such interactions on signal transduction pathways. Emerging evidence indicates asymmetric function of GPCR dimers and communication between the two protomers of the dimer at the level of ligand binding, ligand-induced conformational changes and interaction with intracellular signaling proteins. Negative and positive allosteric interactions between the two orthosteric binding sites of GPCR homo- and heterodimers may have major consequences on the pharmacology and functional drug outcomes.

Although monomeric GPCRs are able to bind and activate G proteins, formation of receptor dimers clearly offers additional opportunities for regulating G protein activation and signal transduction by accommodating the simultaneous binding of other regulatory GIPs to the receptor.

Finally, formation of higher-order oligomeric clusters needs to be considered in the future. Despite the existence of an extensive literature on GPCR dimerization, the precise oligomerization state of GPCRs remains in most cases unknown. Atomic force microscopy studies provided convincing evidence that rhodopsin is organized as long oligomeric arrays of dimers in rod outer segment membranes [45]. This idea was further extended in molecular docking studies based on the resolved structures of rhodopsin and G_t_ by proposing an hexameric complex composed of two G_t_ proteins and a rhodopsin tetramer [46]. These modeling studies obviously warrant experimental confirmation but provide already an interesting framework for the organization and asymmetric binding of different GIPs to GPCR oligomers. Formation of oligomeric complexes might not be restricted to rhodopsin as formation of tetrameric complexes has been proposed for the GABA_B_ receptors, which are composed of two obligatory GABA_B_ heterodimers [47]. Finally, the putative dynamic regulation of GPCR oligomerization might introduce a further level of regulation for GPCR function.
